# The large contribution of twins to neonatal and post-neonatal mortality in The Gambia, a 5-year prospective study

**DOI:** 10.1186/s12887-016-0573-2

**Published:** 2016-03-15

**Authors:** Reiko Miyahara, Momodou Jasseh, Grant Austin Mackenzie, Christian Bottomley, M. Jahangir Hossain, Brian M Greenwood, Umberto D’Alessandro, Anna Roca

**Affiliations:** Medical Research Council, Banjul, The Gambia; Institute of Tropical Medicine, Nagasaki University, Nagasaki, Japan; MRC Tropical Epidemiology Group, London School of Hygiene and Tropical Medicine, London, UK; Faculty of Infectious and Tropical Diseases, London School of Hygiene and Tropical Medicine, London, UK; Murdoch Children Research Institute, Melbourne, Australia; Faculty of Epidemiology and Population Health, London School of Hygiene and Tropical Medicine, London, UK

**Keywords:** Twins, HDSS, Mortality, Risk factors, Neonatal, Post-neonatal

## Abstract

**Background:**

A high twinning rate and an increased risk of mortality among twins contribute to the high burden of infant mortality in Africa. This study examined the contribution of twins to neonatal and post-neonatal mortality in The Gambia, and evaluated factors that contribute to the excess mortality among twins.

**Methods:**

We analysed data from the Basse Health and Demographic Surveillance System (BHDSS) collected from January 2009 to December 2013. Demographic and epidemiological variables were assessed for their association with mortality in different age groups.

**Results:**

We included 32,436 singletons and 1083 twins in the analysis (twining rate 16.7/1000 deliveries). Twins represented 11.8 % of all neonatal deaths and 7.8 % of post-neonatal deaths. Mortality among twins was higher than in singletons [adjusted odds ratio (AOR) 4.33 (95 % CI: 3.09, 6.06) in the neonatal period and 2.61 (95 % CI: 1.85, 3.68) in the post-neonatal period]. Post-neonatal mortality among twins increased in girls (*P* for interaction = 0.064), being born during the dry season (*P* for interaction = 0.030) and lacking access to clean water (*P* for interaction = 0.042).

**Conclusion:**

Mortality among twins makes a significant contribution to the high burden of neonatal and post-neonatal mortality in The Gambia and preventive interventions targeting twins should be prioritized.

## Background

During the past few decades, under-5 year mortality has decreased worldwide, with similar trends in high, middle and low-income countries [1]. In sub-Saharan Africa, under-5 mortality has declined significantly since 2000, although rates are still unacceptably high. The decline in neonatal mortality has been slower than in older children and thus, the relative contribution of neonates to under-5 deaths has increased. In 2013, almost half of under-5 deaths worldwide were neonates [1].

Twins have an increased risk of death during the neonatal period, and this extends at least until the first anniversary. The high rate of mortality in twins is probably due to complications at birth and early life [2–4], including prematurity [5, 6] and low birth weight [7, 8], and cultural beliefs [9] which can influence growth patterns and gender-biased care. Twins require specialist health care in early life, which is often not available in low-income countries.

In West Africa, where health care resources are limited and neonatal and infant mortality are high, the twinning rate (15–18 per 1000 live births) is higher than in other regions such as Eastern Europe (below 9 per 1000 live births) or South and South-East Asia (below 9 per 1000 live births) [10]. A study conducted in The Gambia between 1989 and 1992 showed a twinning rate of 15 per 1000 live births and double the risk of death in this group during infancy [11]. Since 1992, under-five mortality rate has declined in The Gambia by more than 50 %, with a similar decrease among infants (48 %) but there has been less of decline in neonatal mortality (18 %) [12]. The mortality pattern in twins over this period has not yet been documented in the country. To design health interventions that target twins, it is necessary to understand the risk factors for mortality in this group. The aim of this study was therefore to examine excess mortality among twins in The Gambia during the neonatal (within 28 days after birth) and post-neonatal periods (29 days to 365 days) between 2009 and 2013; and to assess epidemiological and demographic risk factors for mortality in this group.

## Methods

### Data source

We used data from the Basse Health and Demographic Surveillance System (BHDSS), which covers the south bank of the Upper River Region of The Gambia and included more than 170,000 individuals during the study period. In the BHDSS, trained field workers visit each household every four months and update demographic events in every household (i.e. pregnancies, births, deaths, in and out migrations). Additional information is transcribed from the antenatal cards and vaccination cards. The procedure is the same as that used in another demographic surveillance site in The Gambia, Farafenni HDSS, and described elsewhere [13].

Socio-economic data were collected in a survey conducted in 2011. The information collected in this survey included: (i) asset ownership (radio, television, video, car, motor cycle, refrigerator, bicycle), (ii) household material (such as roof, wall, floor), and (iii) toilet facility. We developed a socio-economical status (SES) index using theses data by primary component analysis. The SES index was categorized into 5 quintiles from 1^st^ poorest to 5^th^ wealthy [14].

Every pregnancy identified by field workers during demographic update rounds of the HDSS is followed up until termination. Information solicited from the woman on the outcome of the pregnancy include number of children resulting from the pregnancy and the number born alive. Therefore, pregnancies which terminated with two or more children born were classified as multiple births regardless of the number born alive; and all those with only one child born were confirmed as singletons. Deaths during the neonatal and the post-neonatal period were identified during routine household visits.

### Statistical analyses

All children born in the BHDSS from January 2009 to December 2013 were included in the analysis; triplets were excluded. Mortality rates for neonatal and post-neonatal periods were calculated by dividing the number of deaths by the number of live births.

We compared the rate of mortality in twins and singletons using logistic regression to adjust for sex, ethnicity, season of birth, maternal age, birth order, SES index, access to clean water and birth interval (Model 2). Because many children were missing data on SES index, access to clean water and birth intervals, we also conducted an analysis (Model 1) where these variables were excluded from the model. The influence of socio-demographic factors on mortality in twins was compared to their influence in singletons. We used logistic regression to test for effect modification (i.e., different odds ratios in twins and singletons) and adjust for confounding. Confidence interval and *p*-values were computed using cluster-robust variance estimates to adjust for clustering by household. The probability of monozygotic and dizygotic twins were calculated using Weinberg zygosity estimation [15]. All analyses were conducted using Stata version 12.

This study was approved by Gambia Government/Medical Research Council Joint Ethics Committee. Verbal consent of participants of HDSS was obtained by village leaders and individual household heads for household members.

## Results

**Fig. 1:**
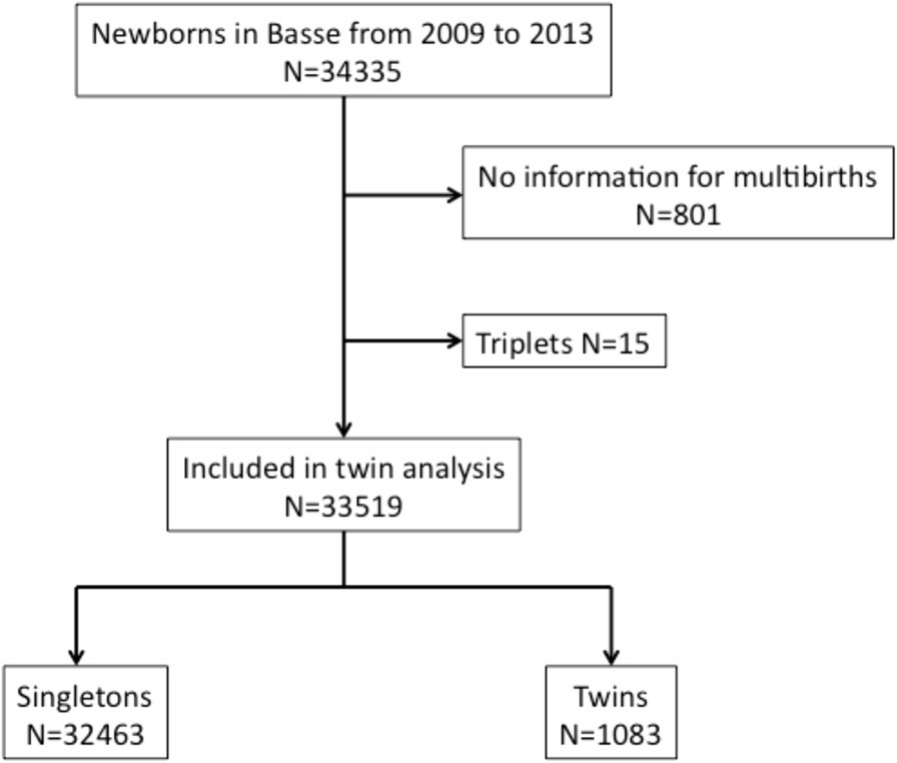
Flowchart of study population in the Basse HDSS, The Gambia, 2009–2013

Between January 2009 and December 2013, a total of 34,335 newborns were registered in the BHDSS. After excluding 801 children without information on multiple birth and 15 triplets, 33,519 children were included in the analysis; 1083 twins (3.2 %) and 32,436 singletons (Fig. 1). The twinning rate was 16.7/1000 deliveries of live births including the 17 deliveries that one still birth in pairs. There were 400 children in boy/girl pairs (37.5 %), 294 in boy pairs (27.6 %) and 372 in girl pairs (34.9 %) among the 1066 study twins with data on gender (98.5 %). Thus, the estimated probabilities of monozygotic and dizygotic twins were 25.0 % and 75.0 %, respectively. Table 1 shows the characteristics of the twins and singletons born during the study period. Compared to singletons, twins were more likely to be female (OR = 1.17, 95 % CI: 1.02, 1.34, *P* = 0.022), to be born in the wet season (OR = 0.79, 95 % CI: 0.66, 0.93, *P* = 0.006), to have mothers older than 25 years of age (OR = 1.95, 95 % CI: 1.62, 2.35, *P* <0.001), and to have mothers who had had at least one previous delivery (OR = 1.40, 95 % CI: 1.17, 1.67, *P* <0.001).

**Table 1:** Characteristics of twins and singletons in the Basse HDSS, The Gambia, 2009–2013 (*N* = 33,519)

		Singletons (*N* = 32,436)		Twins (*N* = 1083)				
		No.	%	No.	%	OR ^a^	95 % CI^b^	*P* value
Sex								
	Boys	16386	50.5	504	46.5	1 (reference)		
	Girls	16049	49.5	579	53.5	1.17	1.02–1.34	0.022
Ethnicity								
	Serahule	14604	45.0	513	47.4	1 (reference)		
	Mandinka	6721	20.7	232	21.4	0.98	0.84–1.15	0.828
	Fula	10508	32.4	321	29.6	0.87	0.75–1.00	0.053
	Others	597	1.8	17	1.6	0.81	0.50–1.32	0.401
Region								
	Rural	28633	88.3	970	89.6	1 (reference)		
	Urban	3803	11.7	113	10.4	0.88	0.67–1.15	0.351
Season of birth ^d^								
	Wet	17294	53.3	641	59.2	1 (reference)		
	Dry	15142	46.7	442	40.8	0.79	0.67–0.93	0.006
Birth order								
	1	14395	44.4	393	36.3	1 (reference)		
	2	12461	38.4	450	41.6	1.32	1.09–1.60	0.005
	3+	5580	17.2	240	12.2	1.58	1.25–1.98	<0.001
Mother age								
	<20	4810	15.0	80	7.5	1 (reference)		
	20–24	10087	31.4	249	23.2	1.48	1.04–2.11	0.024
	25–29	7470	23.3	265	24.7	2.12	1.48–3.04	<0.001
	30–34	5770	18.0	282	26.3	2.94	2.06–4.19	<0.001
	≧35	3978	12.4	196	18.3	2.96	2.04–4.29	<0.001
Mother education level								
	No	5600	17.3	162	15.0	1 (reference)		
	Religious	17201	53.0	633	58.5	1.28	1.00–1.64	0.052
	Basic	2665	8.2	67	6.2	0.87	0.58–1.31	0.517
	Senior	679	2.1	20	1.9	1.02	0.50–2.11	0.948
	Unknown	6291	19.4	201	18.6	1.11	0.82–1.50	0.489
Birth interval (<2 years) ^e^								
	≧2 years	29110	90.2	981	91.6	1 (reference)		
	<2 years	3181	9.9	90	8.4	0.84	0.67–1.05	0.117
Access to clean water ^f^								
	Yes	23723	86.5	793	85.6	1 (reference)		
	No	3700	13.5	133	14.4	1.08	0.82–1.41	0.603
SES index ^g^								
	1^st^/Poorest	5,150	20.3	159	18.6	1 (reference)		
	2nd	6,797	26.8	221	25.8	1.05	0.77–1.43	0.742
	3rd	3,250	12.8	127	14.8	1.27	0.90–1.77	0.180
	4th	5,129	20.2	195	22.8	1.23	0.90–1.67	0.194
	5^th^/wealthy	5,061	19.9	154	18.0	0.99	0.71–1.36	0.930

^a^ Odds Ratio, ^b^ 95 % confidence interval adjusted for clustering by household

^c^ *P* value were tested by Wald test with GEE accounting for clustering by households

^d^ Wet season is from June to October, and Dry season is from November to May

^f^ Clean water is Tap water (Public, in dwelling/compound) and Protected well (*N* = 28,349)

^e^ *N* = 33,362, ^g^ *N* = 26,242

Overall, we recorded 1082 deaths during the follow up period (977 singletons and 105 twins). During the neonatal period, mortality among twins was 55.4 per 1000 live births (95 % CI: 41.8, 69.0) and among singletons 13.8 per 1000 live births (95 % CI: 12.5, 15.1). Twins’ deaths represented 11.8 % of all neonatal deaths and 7.8 % of all post-neonatal deaths (Table 2). Up to sixty percent of twin deaths during the neonatal period occurred within 2 days of birth, compared with 46.2 % of deaths in singletons. Twins had about six times higher risk of death within 2 days after the birth compared to singletons (Adjusted Odds Ratio (AOR) = 5.71, 95 % CI: 3.67, 8.89, *P* < 0.001). The risk of death among twins compared to singletons was higher during the neonatal period (AOR = 4.33, 95 % CI: 3.10, 6.07, *P* <0.001) and post-neonatal period (AOR = 2.61, 95 % CI: 1.85, 3.68, *P* <0.001) (Table 2). Among twins, pairs of concordant and discordant sex showed similar mortality [AOR in neonatal period = 1.07, 95 % CI; 0.60, 1.91 and AOR in post-neonatal period = 0.73, 95 % CI: 0.39, 1.37].

**Table 2:** Mortality rate among twins (*N* = 1083) and singletons (*N* = 32,436) in the neonatal (0–28 days), post-neonatal (29–365 days) period and infancy (0–365 days) in the Basse HDSS, The Gambia, 2009–2013

		No. deaths	Deaths per 1,000 live births	95 % CI ^c^		OR ^a^	95 % CI ^c^	Adjusted OR ^b^ (Model1)	95 % CI ^c^	Adjusted OR ^c^ (Model2)	95 % CI ^d^
Neonatal	All ^e^	508	15.2	13.9	16.5						
	Singletons	448	13.8	12.5	15.1	1 (reference)		1 (reference)		1 (reference)	
	Twins	60	55.4	41.8	69.0	4.19	3.01–5.82	4.33	3.09–6.06	4.86	3.38–6.99
Post neonatal	All ^e^	574	17.1	15.7	18.5						
	Singletons	529	16.3	14.9	17.7	1 (reference)		1 (reference)		1 (reference)	
	Twins	45	41.6	29.7	53.4	2.61	1.86–3.67	2.61	1.85–3.68	2.72	1.84–4.01
Infancy	All ^e^	1082	32.3	30.4	34.2						
	Singletons	977	30.1	28.3	32.0	1 (reference)		1 (reference)		1 (reference)	
	Twins	105	97.0	79.3	114.6	3.46	2.68–4.45	3.51	2.71–4.55	3.83	2.87–5.11

^a^ Odds Ratio

^b^ Odds ratio for twins compared to singletons adjusted for sex, year of birth, ethnicity, season of birth, mother age, birth order (*N* = 33,088)

^c^ Odds ratio for twins compared to singletons adjusted for sex, year of birth, ethnicity, season of birth, mother age, birth order, birth interval, access to water, SES index (*N* = 25,835)

^d^ 95 % confidence interval adjusted for clustering by household

^e^ Singletons and Twins

There was weak evidence that some risk factors for mortality had different effects in twins and singletons only during the post-neonatal period. During this period, the increased risk of mortality among twins was higher in girls than boys (*P* for interaction 0.064), being born during the dry season (*P* for interaction 0.030) and living in a house with lack of access to clean water (*P* for interaction 0.042) (Table 3).

**Table 3:** Risk factors for mortality in singletons and twins in the neonatal (0–28 days) and post-neonatal period (29–365 days) period in the Basse HDSS, The Gambia, 2009–2013

		Singletons (*N* = 32,436)				Twins (*N* = 1083)				
		Univariate model		Multivariate model		Univariate model		Multivariate model		
Neonatal death		OR ^a^	95 % CI ^c^	AOR ^b^	95 % CI ^c^	OR ^a^	95 % CI ^c^	AOR ^b^	95 % CI ^c^	P for Interaction ^d^
Sex	Boys	1 (reference)		1 (reference)		1 (reference)		1 (reference)		0.459
	Girls	0.81	0.67–0.97	0.81	0.67–0.98	0.99	0.58–1.70	1.01	0.59–1.72	
Ethnicity	Serahule	1 (reference)		1 (reference)		1 (reference)		1 (reference)		0.455
	Fula	1.06	0.83–1.35	1.01	0.85–1.39	1.69	0.75–3.84	1.69	0.74–3.83	
	Mandinka	1.11	0.90–1.38	1.13	0.91–1.41	1.20	0.55–2.62	1.21	0.55–2.63	
Season of birth ^e^	Wet	1 (reference)		1 (reference)		1 (reference)		1 (reference)		0.194
	Dry	0.79	0.65–0.95	0.78	0.64–0.94	1.20	0.65–2.21	1.19	0.65–2.21	
Access to clean water ^f^	Yes	1 (reference)		1 (reference)		1 (reference)		1 (reference)		0.231
	No	0.96	0.72–1.29	0.96	0.71–1.28	1.65	0.73–3.73	1.63	0.71–3.75	
Birth order	1	1 (reference)		1 (reference)		1 (reference)		1 (reference)		0.339
	2+	0.82	0.68–0.99	0.79	0.64–0.98	1.15	0.60–2.20	1.01	0.57–2.14	
Birth intervals ^g^	≧2 years	1 (reference)				1 (reference)				0.258
	<2 years	1.06	0.78–1.44	1.24	0.88–1.73	2.03	0.81–5.10	2.15	0.86–5.36	
SES index ^h^	Poor	1 (reference)				1 (reference)				0.236
	Middle	0.80	0.63–1.02	0.80	0.63–1.02	0.55	0.24–1.25	0.57	0.25–1.30	
	Wealthy	0.76	0.57–1.01	0.77	0.56–1.06	0.47	0.15–1.49	0.46	0.14–1.46	
Post neonatal death										
Sex	Boys	1 (reference)		1 (reference)		1 (reference)		1 (reference)		0.064
	Girls	0.96	0.80–1.14	0.95	0.80–1.14	1.78	0.92–3.43	1.81	0.94–3.49	
Ethnicity	Serahule	1 (reference)		1 (reference)		1 (reference)		1 (reference)		0.382
	Fula	1.36	1.08–1.71	1.36	1.08–1.70	0.56	0.24–1.33	0.55	0.23–1.30	
	Mandinka	1.35	1.10–1.65	1.33	1.09–1.63	0.72	0.33–1.59	0.75	0.34–1.65	
Season of birth ^e^	Wet	1 (reference)		1 (reference)		1 (reference)		1 (reference)		0.030
	Dry	0.87	0.73–1.03	0.86	0.72–1.02	1.86	0.95–3.66	1.86	0.95–3.65	
Access to clean water ^f^	Yes	1 (reference)		1 (reference)		1 (reference)		1 (reference)		0.042
	No	0.80	0.59–1.07	0.77	0.57–1.04	1.91	0.84–4.36	1.92	0.84–4.42	
Birth order	1	1 (reference)		1 (reference)		1 (reference)		1 (reference)		0.221
	2+	0.88	0.74–1.04	0.91	0.76–1.10	0.58	0.30–1.14	0.59	0.30–1.16	
Birth intervals ^g^	≧2 years	1 (reference)								0.804
	<2 years	1.22	0.94–1.60	1.42	1.07–1.88	1.09	0.71–0.31	3.91	0.33–4.35	
SES index ^h^	Poor	1 (reference)				1 (reference)				0.182
	Middle	0.77	0.62–0.97	0.80	0.64–1.01	2.15	0.95–4.87	2.14	0.94–4.88	
	Wealthy	0.64	0.48–0.85	0.67	0.49–0.90	1.80	0.57–5.60	1.77	0.57–5.54	

^a^ Odds Ratio

^b^ Odds ratio adjusted for sex, year of birth, ethnicity, season of birth, mother age, birth order

^c^ 95 % confidence interval adjusted for clustering by household

^d^ *P* value for interaction comparing AOR in singletons and twins

^e^ Wet season is from June to October, and Dry season is from November to May

^f^ Clean water is Tap water (Public, in dwelling/compound) and Protected well (*N* = 28,048)

^g^ *N* = 33,362

^h^ Quintiles of SES score grouped: 1&2 poor, 3&4 middle, 5 wealthy (*N* = 25,835)

## Discussion

Our study has shown that mortality among twins remains very high in The Gambia during the neonatal and post-neonatal periods. Because of the high twinning rate, twins contributed to almost 12 and 8 % of neonatal and post-neonatal deaths respectively in the study area. During the neonatal period, mortality among twins was at least four times higher than among singletons, and this higher risk of death continued until the end of infancy. During the post-neonatal period, the excess risk of death among twins increased in girls, among those resident in a house with a lack of access to clean water and among those born during the dry season.

Although overall neonatal and infant mortality has dramatically decreased in The Gambia during the last decades, the high risk of mortality among twins appears not to have fallen substantially. In the present study, the odds of neonatal and post-neonatal mortality were 4.19 and 2.61 times increased in twins, comparable to a previous study in The Gambia, (RR = 6.1 and RR = 2.9 during the early and late neonatal periods and RR = 1.6 during the post-neonatal period) [11]. As neonatal and post-neonatal deaths are relatively rare events, those RR should be comparable to our OR. We found that the increased rate of mortality among twins are similar to that estimated from pooled data from Malawi, Tanzania and Zambia, where the AOR was 6.24 (95 % CI: 5.02, 7.77) for neonatal mortality and 3.05 (95 % CI: 2.36, 3.95) for post-neonatal mortality [16]. In another meta-analysis of 25 demographic health surveys conducted in sub-Saharan Africa, the AOR was 5.55 (95 % CI: 4.26, 7.23) for neonatal mortality and 2.63 (95 % CI: 2.37, 2.90) for post-neonatal mortality [3].

The twinning rate continues to be high in The Gambia (16.7 per 1000 deliveries) compared to the other regions [10] and is similar to that found in a previous study conducted in the country (15 per 1000 live births) [11] and also similar to other West African countries [18 per 1000 deliveries in Guinea-Bissau [8], 18.5 per 1000 deliveries in Nigeria [4]] . Dizygotic twin pregnancies represent more than two thirds of the overall twinning rate in our study as previously shown in the country [11]. The risk factors for twinning that we found in our study are consistent with previous literature in Africa and other regions, which include older maternal age [3], being born during the dry season [17, 18] and having at least one older siblings [19]. As expected, the influence of birth order was diminished after adjusting for maternal age. In contrast to the previous study in The Gambia, which showed a significantly lower twinning rate among Mandinkas (10.4 per 1000 deliveries) [11], we did not observe any significant difference in twinning rate by ethnicity.

We found weak evidence for an increased risk of mortality among girl twins that was higher than among boy twins during the post-neonatal period (*P* for interaction = 0.064). In previous reports from Senegal, mortality among pairs of boy twins was 5.4 times higher (95 % CI: 1.7, 17.0) than pairs of girl twins during the post-neonatal period [20]. Also, pooled Demographic and Health Surveys data from 31 sub-Saharan African countries showed the increased risk of mortality among twins was more marked in boys compared to girls [21]. In general, higher mortality among boys is explained by the biological and immunological reasons [22–25]. Higher mortality among girl twins in post-neonatal period, as we found in our study, has been attributed in other countries to gender discrimination in health seeking behaviour [26] and nutritional status [27].

Season of birth also increased the risk of mortality among twins in post-neonatal period. Among Gambian children, the rainy season increased the risk of low birth weight and as a consequence infant mortality [28–31]. During the post-neonatal period, we have observed an increased risk of mortality in twins being born during the dry season. They should start eating food during the following rainy season when shortage of food is more common. Malnutrition is a more prevalent cause of death among twins than singletons during this age group [11] and therefore, the shortage of food during the post-neonatal period may have a more detrimental effect on twins than singletons. Our findings also indicate that lack of access to clean water during the post-neonatal period increases the risk of death more in twins than singletons. Previous research in Guinea-Bissau [32] and in Malawi [6] showed that twins start complementary food significantly earlier than singletons. If that is the case in The Gambia, it could explain the importance of the lack of access to clean water in increasing the risk of death among twins, as they are exposed from a very early age to contaminated weaning foods which are a risk factor of infectious diseases (especially gastroenteritis which in turn causes malnutrition) [33].

The analysis presented here used the HDSS for evaluating trends on neonatal and post-neonatal mortality among twins. The major strengths of this analysis are the consistency in data collection over the study period and the large denominator that allow detailed comparisons and assessment of risk factors and interactions. Using the HDSS has also important limitations. The BHDSS is updated every four months, and pregnancies and neonatal deaths might have been missed. Furthermore, the twinning rate and twin mortality might have been underestimated if fieldworkers missed neonatal deaths and misclassified the surviving twins as singletons. This may have occurred by the small number of stillbirths that we counted among twins. We systematically collected information on the number of newborns regardless of their status to minimise this potential misclassification.

## Conclusion

Twins are at very high risk of death during the neonatal and post-neonatal periods in The Gambia. Girls, being born in dry season and lack of access to clean water might be associated with increased risk of death among twins during the post-neonatal period. Efforts should be made to identify twin pregnancies during antenatal care visits and to provide regular home visits, maternal education [34] and food supplementation to the women [31] before and after delivery.

## Abbreviations

HDSS: health and demographic surveillance system
OR: odds ratio
AOR: adjusted odds ratio
SES: socio economic status
